# Column Bioleaching of Fluoride-Containing Secondary Copper Sulfide Ores: Experiments With *Sulfobacillus thermosulfidooxidans*

**DOI:** 10.3389/fbioe.2018.00183

**Published:** 2019-02-18

**Authors:** Michael L. M. Rodrigues, Guilherme H. A. Santos, Hamilton C. Leôncio, Versiane A. Leão

**Affiliations:** Bio&Hydrometallurgy Laboratory, Department of Metallurgical and Materials Engineering, Universidade Federal de Ouro Preto, Ouro Preto, Brazil

**Keywords:** bioleaching, *Sulfobacillus thermosulfidooxidans*, aluminum, fluoride, secondary copper sulfides, columns

## Abstract

Bioleaching is a mature technology, which is widely employed commercially in the leaching of primary sources of metals (ores, concentrates, and mine residues). The current work discussed the effects of aluminum sulfate additions to the growth medium, PLS recirculation and bleeding on the column bioleaching of secondary copper sulfide ores with a significant content of fluoride-containing minerals. Fluoride is toxic to bacteria at the pH of bioleaching but its toxicity may be overcome in the presence of soluble aluminum and ferric iron. Therefore, experiments were carried out in 10 × 100 cm height aerated columns, loaded with 10 kg of crushed and agglomerated copper ore and inoculated with *Sulfobacillus thermosulfidooxidans*. Initially, fluoride concentrations of up to 2.5 g/L in the pregnant leach solution were observed due to the fast dissolution of fluoride-bearing minerals. Aluminum was added to the leaching solution to reduce the Al/F ratio so that the concentration of HF (the main toxic species) was decreased, but while the total fluoride concentration was higher than that of aluminum, the bacterial population as low. Therefore, the current work emphasizes that it is possible to set up conditions to enable bioleaching even at high fluoride concentrations. Following this approach, copper extractions above 90% were achieved for a H_2_SO_4_ consumption ranging from 128.8 to 206.1 Kg/ton.

## Introduction

Mining companies constantly search for new process routes to treat low-grade ores and metal–bearing mining residues. Bioleaching can be cited as one of the technologies to treat such kind of materials. The discovery and application of microorganisms, which can produce ferric iron and protons (depending on the mineral) either in solution or the mineral surface provided several advances in terms of leaching. Currently, such a technology is already integrated into general leaching processes, and the term bioleaching is now associated with hydrometallurgical practice for sulfides, especially in heap operations.

Copper bioleaching particularly in heap operations has received widely attention, which resulted in the industrial processing of secondary copper sulfides, especially in the case of chalcocite (Cu_2_S) ores. Heap leaching was initially used to replace vat leaching in the processing of copper oxide ores. With its conversion to treat secondary sulfides in the presence of microorganisms and along with solvent extraction and electrowinning, it became a major breakthrough in the copper industry and currently accounts for nearly around 15% of the overall metal production worldwide, particularly for low and moderate copper grades (Acevedo and Gentina, 2013).

Sulfide oxidation is exothermic process and depending on the heat releasing rate or in warm areas of the planet the temperature in the heaps may reach a point which enables the growth of moderate thermophiles. This was demonstrated in the escondida mine operation (Chile), when the third lift operation was started and resulted in a change from mesophiles to almost 100% of the 16S rRNA copies as *S. thermosulfidooxidans* (Soto et al., 2013). Both temperature and the type of microorganism can affect copper extraction from secondary ores. For instance, experiment carried out with an ore containing 80% covellite inoculated with the genera *Acidianus* and *Metallosphaera* resulted in a 75% copper extraction, at 65°C, in 345 days. A column containing the same ore, but inoculated with *At. ferrooxidans* and *Leptospirillum ferrooxidans* (at 20–23°C) presented a copper extraction below 20% (Acar et al., 2005). Lee et al. (2011) compared the bioleaching performance of ore samples containing either chalcocite, covellite, or enargite as the predominant sulfide. The chalcocite-rich sample was bioleached by both mesophilic and thermophilic strains with copper extractions ranging from 89.9% to 99.2%. The covellite-bearing columns were leached by thermophiles resulting in an extraction of 88.3–95.4%, in 300 days, however copper extraction was lower than 20% in a 240-days period under mesophilic conditions. These results were consistent with another study on mixed-sulfide bioleaching, which reported slower covellite dissolution as compared to that of chalcocite (Olson and Clark, 2001).

Fluoride and other anionic species dissolved during bioleaching may have deleterious effects on microbial growth and bioleaching. Razzell and Trussell (1963) reported that 7.6 mg/L fluoride caused a 30% reduction in the Fe^2+^ oxidation rate by *At. ferrooxidans*, whereas complete inhibition was observed for 30 mg/L of fluoride. Both bacterial growth and chalcopyrite bioleaching were impaired by 0.3 g/L total fluoride, according to Dopson et al. (2008). Subsequently, Brierley and Kuhn (2010) studied chalcocite bioleaching with *At. ferrooxidans* and pointed out that the presence of Fluoride-containing minerals completely inhibited copper bioleaching due to concentrations as high as 15 g/L in the pregnant leach solution (PLS).

Previous investigations into the bioleaching of fluoride-bearing copper sulfide ores clearly indicated the necessity of complexing most of the fluoride released during batch experiments (Brierley and Kuhn, 2010). Fluoride was complexed by aluminum additions (as Al_2_(SO_4_)_3_) to the growth medium in concentrations not harmful to bacterial growth, but capable of significantly reducing the free fluoride content (Sicupira et al., 2011; Veloso et al., 2012). Notwithstanding, it was not possible to anticipate the effects of parameters, such as solution recirculation and bleeding on the leaching chemistry (particularly Al^3+^, Fe^3+^, and F^−^ concentrations) as well as copper extraction from such high-fluoride ores. Therefore, the goal of the current paper is to show that the presence of soluble fluoride is not an issue in itself; it is the chemistry of the solution which will define the extent of the impact of fluoride on bioleaching.

## Experimental

### Ore Samples

Bioleaching experiments were carried out with two secondary sulfide ore samples, whose chemical analysis is listed in Table 1. Henceforth these samples will be referred to as high-grade (HG) and low-grade ores (LG).

**Table 1:** Main elements analyzed in the two ore samples.

**Sample**	**Assay (%)**						
	**Cu**	**Al**	**Fe**	**Mg**	**Ca**	**F**	**Cl**
Low-grade ore	1.11 ± 0.03	4.06 ± 0.19	30.73 ± 0.98	1.46 ± 0.04	3.58 ± 0.21	0.65 ± 0.06	0.58 ± 0.12
High-grade ore	1.80 ± 0.04	3.21 ± 0.15	33.39 ± 1.02	1.37 ± 0.06	3.64 ± 0.17	0.71 ± 0.22	0.61 ± 0.14

**Figure 1:**
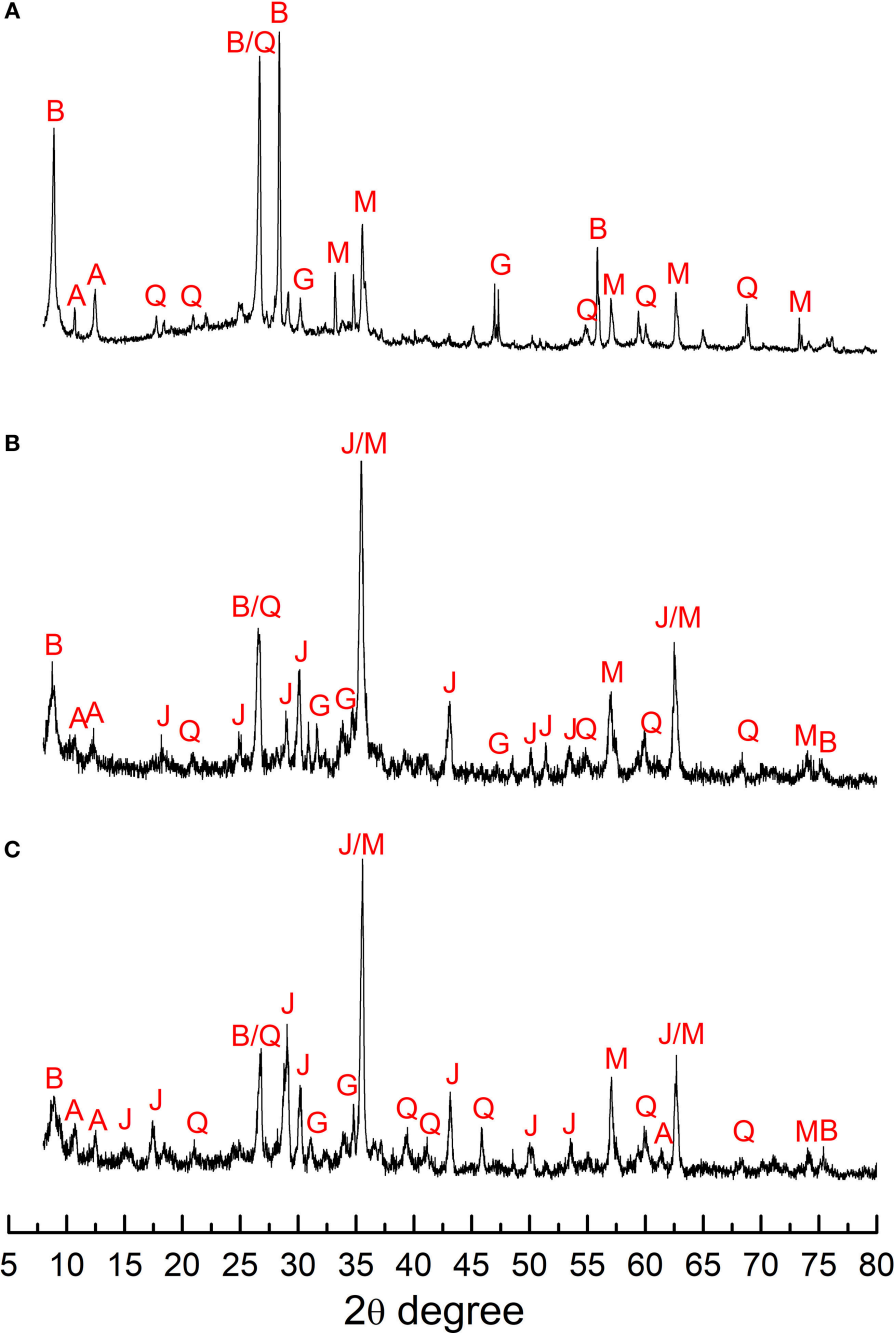
XRD analysis of the original sample **(A)** and the leaching residues of the non-inoculated **(B)** and reference **(C)** columns. Experiments with the high-grade ore. A, grunerite or greenalite; B, biotite; J, K-jarosite; Q, quartz; G, garnet; M, magnetite.

Mineralogical analysis performed by XRD (Figure 1), optical microscopy as well as SEM-EDS indicated that both ores have significant content of fluorite (Table 2), which is soluble in acid media. Moreover, the high-grade copper ore sample contained mostly biotite and magnetite, followed by silicates, whereas the low-grade copper ore presented less biotite and magnetite as well as more garnet. The copper containing minerals in the high-grade sample comprised bornite, which represented 36% of the copper sulfides, along with chalcocite (64%). On the other hand, the low-grade ore assayed 39% bornite, 55% chalcocite, and 6% chalcopyrite. In both cases, cyanide-soluble copper accounted for 92.2% of the total copper in the high-grade ore and 85.0% in the low-copper ore, which is consistent with the mineralogical characterization. Both ores also contained 0.58–0.61% chloride.

**Table 2:** Main minerals analyzed in the two ore samples.

**Sample**	**Minerals (%)**							
	**Biotite**	**Magnetite**	**Amphibole**	**Garnet**	**Fluorite**	**Copper sulfides**	**Quartz**	**Others**
Low-grade ore	34.9	9.5	25.2	16.7	0.7	1.1	4.1	7.8
High-grade ore	42.3	21.5	18.9	6.9	1.2	1.5	2.4	5.3

### Bioleaching Experiments

The bioleaching experiments were carried out in five 100 cm-height × 10 cm-diameter water jacketed fiberglass columns and maintained at 50 ± 1°C. One extra column was operated at room temperature. Each column was loaded with the copper ore samples (~10 Kg) crushed to a ¡″ top size and previously agglomerated with sulphuric acid (10 Kg/ton). All columns were fed by the top from 10-L containers at a flow rate of 1 mL/min (8.6 L/m^2^·h), which was controlled by peristaltic pumps. Oxygen (air) supply was provided to each column by oil-free compressors at a flow rate of 250 mL-air (STP)/min [2.16 m^3^-air(STP)/m^2^/h] and fed at the column base.

A *Sulfobacillus thermosulfidooxidans* strain was supplied the Deutsche Sammlung von Mikrorganismen und Zellkulturen collection (DSMZ 9293). This strain was maintained in Norris growth medium (0.4 g/L (NH_4_)_2_SO_4_, 0.8 g/L MgSO_4_·7H_2_O and 0.4 g/L K_2_HPO_4_) supplemented with yeast extract (0.1 g/L), at 50°C and 150 min^−1^. Ferrous iron (1.0 g/L) was utilized as growth substrate. Prior to column inoculation, this strain was adapted to both ore samples in 2.5% (w/v) solid content batch experiments in the presence of Norris medium. Growth was replicated until 1 L of a solution containing 10^7^ cell/mL was produced, which was utilized for column inoculation.

During the bioleaching experiments, the same Norris growth medium was used for growth although no external ferrous sulfate was supplied to the columns so that the ores provided all iron in solution. Apart from the No-Al column, the Norris growth medium was amended with aluminum sulfate so that at least 1 g/L Al^3+^ was present in the inlet solution during inoculation onwards.

The set of four inoculated columns depicted in Table 3 was inoculated in the 12° day of experiment with *S. thermosulfidooxidans* pre-adapted to the ore as previously described. Both the uninoculated and inoculated experiments run for a period of nearly 250 days, but only results related to the first 100 days of operation are presented herein unless otherwise stated.

**Table 3:** Experimental conditions utilized in the bioleaching study.

**Column**	**Type of ore**	**Temp (^**°**^C)**	**Inoculated**	**Al^**3+**^ added (g/L)**	**Type of loop**	**Legend**
C1	Low-grade	50	No	1.0	Closed	LG ch. leaching
C2	Low-grade	20–25	Yes	1.0	Closed	LG low temp
C3	High-grade	50	No	1.0	Closed	HG ch. leaching
C4	High-grade	50	Yes	1.0	Open	HG open loop
C5	High-grade	50	Yes	–	Closed	HG No Al
C6	High-grade	50	Yes	1.0	Closed	HG Reference

The pregnant leach solution (PLS) was collected from the base of each column and liquor aliquots where withdrawn weekly for subsequent analysis (pH, Eh bacterial counts and metal concentration). Once a week, the PLS volume was recorded and used in copper extraction calculations whereas evaporation losses were compensated with distilled water. The solution was recirculated after the pH was adjusted with concentrated H_2_SO_4_ (closed-loop test), whereas the PLS was discharged in the open-loop experiments. The pH of the leach solution inside each column was corrected to 1.7 ± 0.1 by independently varying the inlet pH, weekly. Solution bleedings were carried out whenever the copper concentration in the PLS exceeded 4 g/L (at the 41st, 61st, 82nd, 152nd days). Table 3 depicts the main operational parameters applied in the six columns studied. The reference condition stands for inoculated column, external aluminum addition at inoculation (1.0 g/L), closed loop, and the high-grade ore.

### Analytical and Characterization Techniques

Metal concentrations in the PLS were assessed by ICP-OES, Varian 725, whereas fluoride was determined by ionic chromatography (Metrohm) utilizing a conductivity detector. Measurements of pH and Eh were carried out in a digital pH/mV meter (DIGIMED, DM-20 model) with a Ag/AgCl reference electrode (for Eh) and combined DMR-CP1 platinum electrode (for pH). Acid consumption was determined from the mass of sulphuric acid utilized to correct the pH of the leaching solution. Cell counts were performed using a Neubauer chamber in a phase contrast microscope (Leica). The ferrous iron concentration was determined by titration with standard potassium dichromate solution in the presence of a 1 H_2_SO_4_: 1 H_3_PO_4_ solution using an automatic titrator (Schott—Tritoline Alpha).

The mineral phases in both the initial samples and in the residues were identified by the powder method in a X-ray diffractomer (Shimadzu XRD 6100). This device was equipped with a copper tube and operated at 40 kV and 30 mA. Scanning was taken in the 2θ range between 8 and 70° with steps of 0.02°/2θ and a scanning rate of 2°/min.

## Results

### Copper Extraction, pH Profile, and Acid Consumption

The bioleaching of secondary copper sulfides (reactions 1 and 2) relies on the ferric iron production either in the bulk of the solution (the indirect mechanism) or in a biofilm on the mineral surface (the indirect-contact mechanism). Also, the bacteria must oxidize all the elemental sulfur produced during the Fe^3+^ attack on the mineral surface so that full metal extraction is achieved. These bacteria obtain their energy, primarily by oxidation of ferrous ion and reduced sulfur compounds (Sand et al., 2001; Watling, 2006). As the SEM analyses of bioleaching residues showed the absence of elemental sulfur (a product of chalcocite oxidation) it was assumed that the element was bio-oxidized during bioleaching in the current work.

(1) $$\begin{array}{rcl} \text{C}{\text{u}}_{2}\text{S}+4\text{F}{\text{e}}^{3+} & \to & 2\text{C}{\text{u}}^{2+}+4\text{F}{\text{e}}^{2+}+{\text{S}}^{0} \end{array}$$

(2) $$\begin{array}{rcl} \text{C}{\text{u}}_{5}\text{Fe}{\text{S}}_{4}+12\text{F}{\text{e}}^{3+} & \to & 5\text{C}{\text{u}}^{2+}+13\text{F}{\text{e}}^{2+}+4{\text{S}}^{0} \end{array}$$

**Figure 2:**
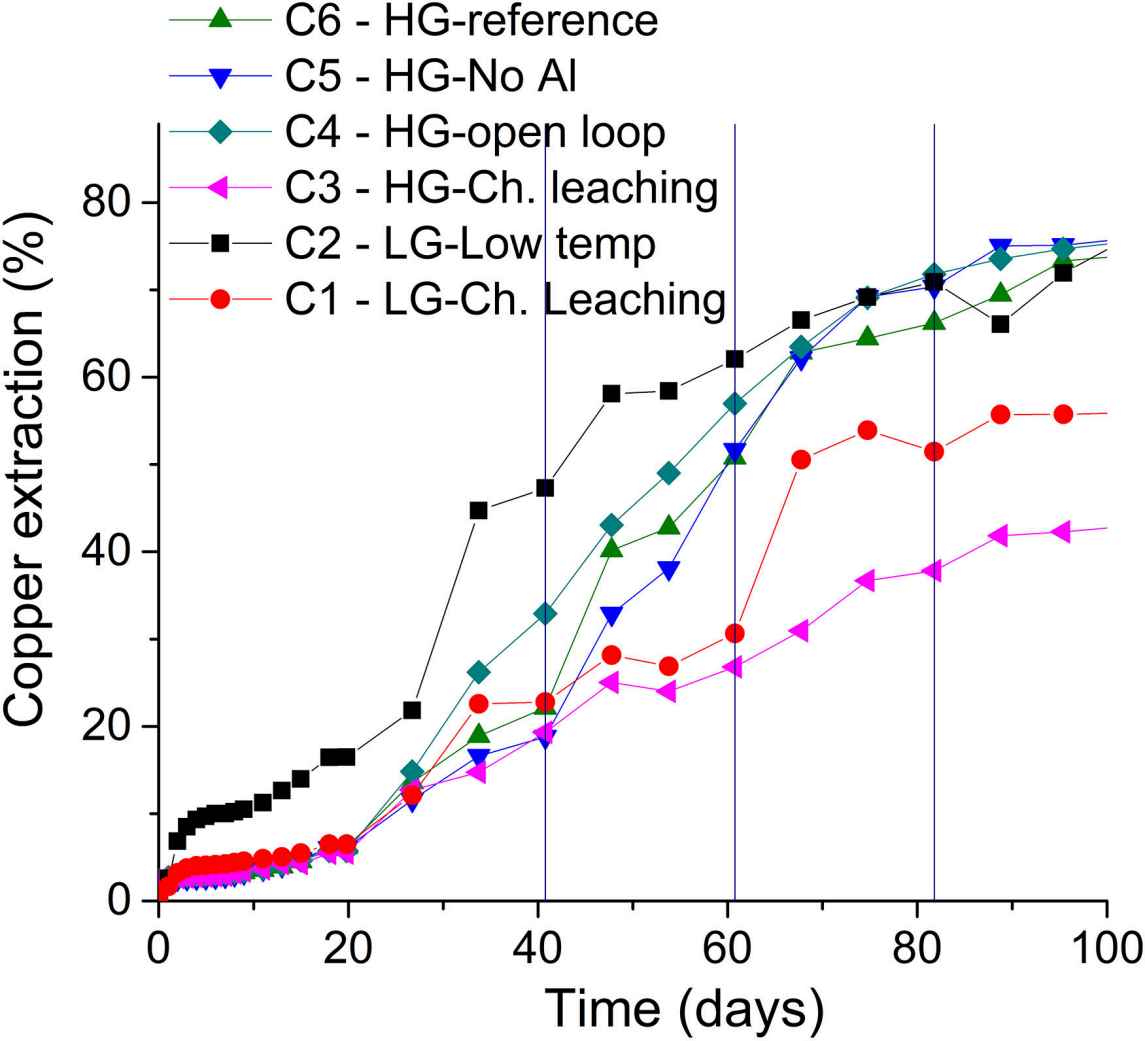
Copper extraction from the high-grade (HG) and low-grade ores (LG). The vertical lines indicate solution bleeding. Experiments with *S. thermosulfidooxidans* growing in Norris growth medium supplemented with yeast extract (0.1 g/L) and amended with 1.0 g/L Al^3+^ (unless otherwise stated); at 50 ± 1°C (columns C1, C3–C6) or room temperature (column C2); crushed and agglomerated ore (¡″ top size); 8.6 L/m^2^/h solution flow-rate and 2.16 m^3^ air/m^2^/h air flow-rate.

**Figure 3:**
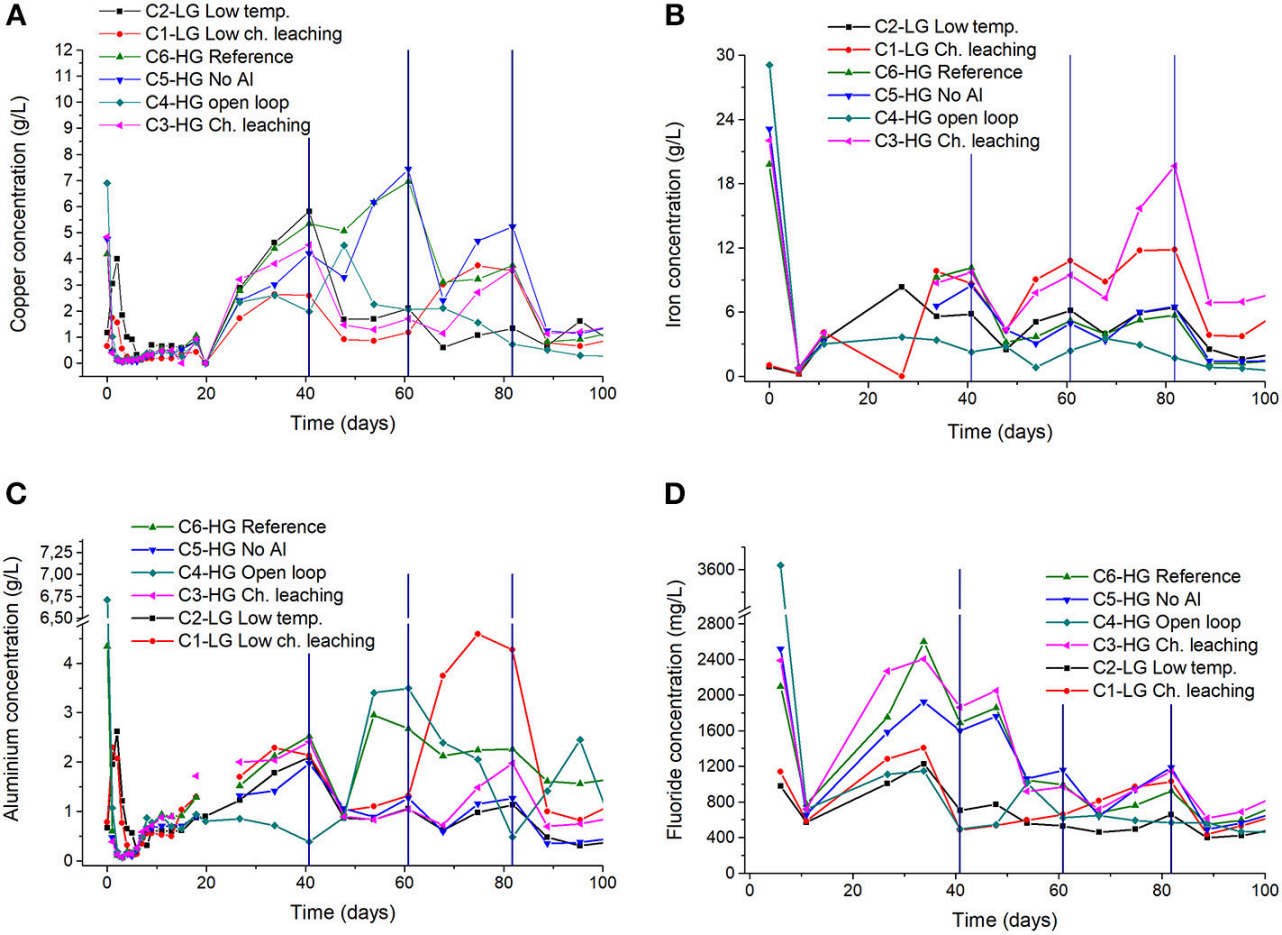
Copper **(A)** iron **(B)** aluminium **(C)** and fluoride **(D)** concentration in the PLS for high-grade ore (HG) and marginal-grade ore (LG). The vertical lines indicate solution bleeding. Experiments with *S. thermosulfidooxidans* growing in Norris growth medium supplemented with yeast extract (0.1 g/L) and amended with 1.0 g/L Al3^3+^ (unless otherwise stated); at 50 ± 1°C (columns C1, C3–C6) or room temperature (column C2).

Figure 2 depicts the dissolution of the low-grade (LG) and high-grade (HG) secondary copper sulfide ores in acid medium as indicated by the copper extractions observed. The analysis of the experiments carried out with the high-grade ore (HG) shows similar leaching kinetics in the case of all inoculated columns (C4–C6), which were both faster and larger than that achieved in the non-inoculated column (C3), particularly after the first solution bleeding (when copper concentration attained 4 g/L (Figure 3A). It is important to highlight that the high concentrations of copper iron aluminum and fluoride were a result of the ore agglomeration with sulphuric acid (10 Kg/ton), which reacted with the minerals and dissolved such elements. Therefore, copper extractions reached 80% within 100 days in the inoculated experiments with the high grade ore as compared to 40% in the non-inoculated column (C3). Similar behavior was observed for the low-grade ore although copper extraction in the non-inoculated column was higher (60%). It must be stressed that secondary sulfides are acid soluble and fairly good extractions are expected regardless of the presence of bacteria (Brierley and Kuhn, 2010). However, the current work shows that bacteria increase the leaching kinetics.

No significant difference in copper leaching from the No-Al (C5) and the reference (C6) columns was observed, suggesting that aluminum additions to growth medium was not required from the copper extraction perspective. The outcomes of the current work were consistent with the findings of Manafi et al. (2013), who investigated the column bioleaching of a porphyry copper sulfide ore containing pyrite grains coated with chalcocite. Recent studies into column bioleaching of a polymetallic sulfide ore containing 125 μg/g Cu reported 70% extraction from an ore assaying 40–60 wt% of acid-soluble copper at both 35 and 50°C, working in the 1.2–2.0 pH range (Watling et al., 2014).

Each bioleaching microorganism has an optimum pH range for growth, whose lower limit is ~1.2 for *S. thermosulfidooxidans* and jarosite precipitation sets the maximum working pH at 2.0–2.2. Therefore, the pH value of 1.7 ± 0.1 was selected for the experiments with this moderate thermophile strain. That was accomplished by setting the pH of the inlet solution at 1.25–1.75 during inoculation (12° day of experiment onwards), which had to be subsequently reduced to the 1.0–1.6 range, because pH values near to 3.5–4.0 were been recorded in all columns during this period. This outcome was a result of acid attack on the readily-soluble minerals present in the ore. Values of pH above 3.0 continued to be registered in the following 30 days of testing, but the dissolution of acid-consuming minerals slowed afterwards and resulted in a reduced scatter in pH readings (PLS), which varied between 1.6 and 1.8 until conclusion of the experiments.

The sulphuric acid consumption required for maintaining the pH at 1.7 ± 0.1 inside the columns varied from 123.3 Kg/ton (column C2) to 206.1 kg/ton (column C3) (Table 4). The non-inoculated columns (C1 and C3) presented the largest acid consumption, due to a lack of jarosite precipitation as suggested by the lowest acid consumption (123–132 kg/ton) depicted by the inoculated closed-loop columns. This outcome emphasized the positive effect of presence of bacteria on acid consumption. Furthermore, the largest acid consumption rate was recorded during the first 75 days, approximately, regardless of the temperature applied. This high rate matched the period in which the input pH was maintained at its lowest value (1.0) and that also represented the period of the fastest metal extraction (Figure 2). The profiles of the main elements solubilized from the gangue minerals and their effect on copper extraction will be further discussed in the current study.

**Table 4:** Important parameters recorded during column bioleaching of secondary sulfide ores.

**Parameter**	**C2-LG low temp**.	**C1-LG Ch. leaching**	**C6-HG reference**	**C5-HG No Al**	**C4-HG open loop**	**C3-HG Ch. leaching**
Acid consumption (Kg/ton)^*^	123.3	176.0	133.0	128.9	164.3	206.1
Solution potential (mV)	655.6	393.8	672.3	637.0	643.3	392.0
Bacterial population (cells/mL)	1.1E+06	n.d.	1.1E+06	3.0E+05	2.1E+06	n.d.
Cu extraction based on the residue (%)^*^	80.4	78.9	92.5	90.0	91.0	73.3
Al extraction based on the PLS (%)^*^	10.7	21.2	31.0	17.6	49.4	20.9

* *After 225 days of experiment*.

### Cell Counts, Solution Potential, and Iron Profile

The inoculation procedure comprised feeding each inoculated experiment (columns C2, C4–C6) with 1 L of a solution containing 10^7^ cells/mL and a redox potential ~700 mV. Such an approach resulted in bacterial populations between 10^5^ and 10^6^ cells/mL in the PLS of the inoculated columns on the day immediately after inoculation (12th day), which remaining in this range throughout the experiments (Table 4). These findings are consistent with the work of Halinen et al. (2009a), who reported bacterial counts ~10^5^ cells/mL in an experiment with *Sulfobacillus thermotolerans* as the predominant strain. Similarly, Watling et al. (2014) reported 10^4^ cells/mL in a PLS containing high aluminum (29–31 g/L), iron (~11 g/L) and magnesium (~12 g/L) concentrations. In the current work, actual populations might have been higher because the containers, which collected the PLS were not heated and cell mobility was reduced as temperature decreased, thus affecting the counting process. Comparing the whole column set, the lower bacterial counts were observed in the column without external Al_2_(SO_4_)_3_ additions (C5), which will be discussed further in the current work.

The solution potential is an indication of bacterial activity in the system, and Table 4 shows high Eh values in all inoculated columns. The value of 550 mV was surpassed only after the first solution bleeding (41st day) in the case of the high grade ore (C4–C6). Prior to that, similar copper extractions were observed in both inoculated and non-inoculated columns (Figure 2) and may be resulted from ferric iron reduction during sulfide oxidation. The high solution potentials (600 mV to 700 mV, regardless of the ore type) observed in the inoculated columns were a result of a rapid production of Fe^3+^ ions by *S. thermosulfidooxidans* (Watling, 2006).

In a bioleaching context, the solution potential is defined by ferrous and ferric iron activities in the PLS; thus, the Fe_tot_ profile is shown in Figure 3B. The inoculated experiments (C2, C4–C6) presented lower soluble iron concentrations than the non-inoculated columns because the presence of *S. thermosulfidooxidans* resulted in the predominance of ferric iron (i.e., high Eh), which along with the high temperature (50°C) induced jarosite precipitation. Any extraction calculated from iron concentrations in solution were thus affected by this precipitation e. g. extractions were lower (< 10%) than those observed in the non-inoculated columns (18%), in which ferrous iron prevailed. Maximum concentrations of up to 16 g/L iron (as Fe^2+^) were observed in the non-inoculated columns (C1 and C3) containing both ore samples, whereas the figure reduced to 6–8 g/L, in the inoculated columns operating at 50°C. Therefore, the results from the non-inoculated columns should be taken as measurement of iron extraction in the whole system because there was no ferrous iron addition to the leach solutions and no relevant ferric iron precipitation.

### Dissolution of Gangue Elements—Liquor Chemistry

In addition to iron, the low pH values applied in the experiments resulted in the release of other elements, which are important for bioleaching processes. Thus, from a metallurgical and bacterial viewpoint, the profile of the most critical species must be assessed. The presence of Al^3+^ ions in solution was a result of the dissolution of silicate minerals present in both ores and also from external aluminum additions [Al_2_(SO_4_)_3_] to the system. Aluminum sulfate was added to ensure a concentration of at least 1.0 g/L Al^3+^ during inoculation and onwards, because a previous investigation (Sicupira et al., 2011) revealed fluoride in the PLS, which had a serious detrimental effect on bacterial growth. Aluminum forms strong complexes with fluoride and reduces the HF concentration as will be discussed further.

The aluminum content added to the experiment was considered in the analysis of metal extractions and Table 4 shows that the largest extraction (49.4%) was observed in the open loop column (C4). The lowest extraction was observed for the low temperature column, which is agreement with the acid consumption data (Table 4). Analyzing the aluminum concentration, the highest values with peaks close to 4 g/L (Figure 3C) were observed between the 50th and 80th days, which implies in a slower dissolution kinetics of aluminum-containing minerals. Any deleterious effects of the presence of aluminum (< 5 g/L) on the ionic strength of the solution and hence bacterial growth were not expected because the aluminum concentration must reach values above 10 g/L to affect Fe^2+^ bio-oxidation by *S. thermosulfidooxidans* (Sicupira et al., 2011). For instance, Watling et al. (2014) justified the low bacterial populations of an industrial heap by aluminum concentrations of up to 30 g/L observed in the PLS.

In addition to aluminum, magnesium and calcium were analyzed and their dissolution were also related to acid consumption, but no detrimental effects on bacterial growth were expected. Actually magnesium is an important nutrient for bacterial growth and is present in the growth medium. The maximum magnesium concentrations attained ~800 mg/L (20% dissolution), whereas calcium concentrations, ~700 mg/L, were controlled by gypsum solubility as also reported by Watling et al. (2014). From the open circuit columns (unsaturated with respect to gypsum), ~25% calcium dissolution was estimated. These figures are similar to that presented by Halinen et al. (2009b), who measured 1.2 g/L magnesium and 0.57 g/L calcium during the bioleaching of low-grade black schist ore.

Chloride and fluoride, which were detected during the mineralogical characterization, were also present in the PLS and both anions are harmful to the bacteria (Dopson et al., 2008). However, chloride concentrations were mostly below 300 mg/L, which were not expected to impair bioleaching (Suzuki et al., 1999). On the other hand, fluoride concentrations as high as 2.5 g/L were recorded (Figure 3D) in the first 50 days, but they were reduced to < 800 mg/L within 100 days because of solution bleedings. The presence of such an element in PLS is related to the presence of fluorite and biotite mostly in the high copper ore (1.2 and 42.3%, respectively) as compared to the low-grade ore (0.7 and 34.9%, respectively).

### Analysis of the Bioleaching Residue

High values of acid consumption are related to the dissolution of Mg-, Ca-, and Al-bearing gangue minerals. Biotite specifically is a fluoride-bearing silicate, which readily dissolves in acid environments (equation 3) resulting in anionic concentrations in the PLS capable of either impairing Fe^2+^ bio-oxidation (Dopson et al., 2008) or completely inhibit bioleaching (Brierley and Kuhn, 2010). This silicate dissolution was a purely chemical process, which was not affected by the presence of microorganisms, but was influenced by temperature.

$$\begin{array}{r} {\text{K}}_{2}\left[\text{S}{\text{i}}_{6}\text{A}{\text{l}}_{2}\right]\text{M}{\text{g}}_{4}\text{F}{\text{e}}_{2}O_{20}{\left(\text{OH}\right)}_{4}+10{\text{H}}^{+}+6{\text{H}}_{2}\text{O}+1/2O_{2} \end{array}$$

(3) $$\begin{array}{r} \to \text{A}{\text{l}}_{2}\text{S}{\text{i}}_{2}{\text{O}}_{5}{\left(\text{OH}\right)}_{4}+\text{Fe}{\left(\text{OH}\right)}_{3}+4{\text{H}}_{4}\text{Si}{\text{O}}_{4}+2{\text{K}}^{+} \end{array}$$

The acid consumption observed herein for all columns was similar to values reported elsewhere (Dopson et al., 2008). Figure 1 shows an XRD analysis of the bioleaching residue of the reference (inoculated) and the chemical leaching (uninoculated) columns (C6 and C3, respectively) and revealed the presence of magnetite potassium jarosite, quartz, biotite and garnet. Although biotite (42%) and magnetite (21%) were the main minerals in the high-grade ore, Figure 1 shows jarosite as the main phases in the residues of the uninoculated (C3) and inoculated (C6) columns, suggesting that the biotite content was significantly reduced during the experiment. Therefore, biotite dissolution accounted for the large acid consumption values observed in the current work (Table 4). Its dissolution was probably larger during the period when the inlet pH was 1.0 because higher acidities have an important contribution to the leaching of such mineral (Kalinowski and Schweda, 1996; Dopson et al., 2008; Bhatti et al., 2012). Similar XRD patterns were observed for the remaining columns, confirming that the biotite dissolution was not related to presence of *S. thermosulfidooxidans*, as expected and was also the main mineral accounting for the aluminum concentrations in the pregnant leaching solution.

Interlayer potassium ions were also released during biotite dissolution (Kalinowski and Schweda, 1996) and the ions precipitated subsequently as potassium jarosite. This is evidenced in Figure 1 which shows the presence of potassium jarosite in the residue. Figure 1 also shows the presence of magnetite in the residue, suggesting that biotite also contributed for most of the iron released from the ore. Soluble iron was subsequently oxidized and also precipitated as jarosite in the inoculated experiments.

## Discussion

The presence of *S. thermosulfidooxidans* in the inoculated columns was previously confirmed using RFLP and PCR-DGGE techniques. Although the presence of other iron oxidizers cannot be ruled out, especially for the column which operated at room temperature (C2), the microbial diversity was low as indicated by PCR-DGGE (Rodrigues, 2012) and was consistent with previously published studies (Cheng et al., 2009; Watling et al., 2014). An indirect analysis of the growth of different *Sulfobacillus* strains was carried out by Watling et al. (2008) who reported ferrous iron oxidation, at a reduced rate, at temperatures below 50°C, which might explain the findings described herein.

The iron profile is important in bio-hydrometallurgical operations because Fe^2+^ is a substrate for bacterial growth (Sand et al., 2001). Nevertheless, both pH and temperature play a key role on iron concentration (and thus Eh values) due to ferric iron precipitation as jarosite. Although pH values below 2.0 are recommended to prevent jarosite formation (Pina et al., 2010), its precipitation is kinetic controlled, thus at high temperatures, such as those applied during leaching with moderate thermophiles the precipitation rate is increased (Daoud and Karamanev, 2006; Ozkaya et al., 2007; van Hille et al., 2010). Hence, jarosite precipitation was significant even at pH below 2.0 in the current work, thus controlling iron solubility and explaining the above-mentioned results (Figure 3B). Focusing on the inoculated closed-loop columns iron concentrations were the largest in column C5 (no aluminum sulfate addition; Figure 3B), in which bacterial populations (Table 4) and thus aluminum concentrations (Figure 3C) were the lowest. A similar outcome was observed in column C2, which operated at lower temperature wherein jarosite precipitation was kinetically hampered. Nevertheless, jarosite was easily noticed in the residues of all inoculated columns irrespective of the temperature investigated. Jarosite precipitation (which also produces acidity) also explains the lower acid consumption (Table 4) observed in the inoculated columns operating at 50°C.

Particularly in the beginning of the experiments, all Fe^3+^ produced by the bacteria was quickly consumed in the leaching of the secondary sulfides according reactions 1 and 2. Such dissolution can be explained by the indirect contact mechanism, i. e. the action of a Fe^3+^-rich biofilm on the surface of the ore particles (Rohwerder et al., 2003). The adsorbed Fe^3+^ has a key role on mineral dissolution during column leaching, but does not affect the values of solution potential (Sand et al., 2001). Nevertheless, increased Eh values were eventually recorded, as copper dissolution progressed and the amount of sulfide was reduced implying in ferrous iron oxidation in the bulk solution, the so-called indirect non-contact mechanism.

Fluoride is toxic to bacteria because it is present mainly as HF (pKa = 3.2, at 25°C and I → 0), at the pH values found in bioleaching processes (pH 1.5–2.0). HF is sufficiently small to penetrate cell membranes, where pH is neutral, whereupon HF dissociates into H^+^ and F^−^, increasing the acidity. This results in inhibition of microbial growth and thus impairs ferrous iron oxidation (Veloso et al., 2012). In acid solutions, Al^3+^ and also Fe^3+^ form highly stable complexes with fluoride (Martel and Smith, 2003) so that both HF concentration and its toxicity are reduced. Because ferric iron is always present during bioleaching with moderate thermophiles, it would reduce the deleterious effects of fluoride, but ferric iron had to be produced by the bacteria prior to effectively complexing F^−^ ions. Because iron dissolves from the ore as Fe^2+^ and free fluoride reduces bacterial growth even at low concentrations, an insufficient amount of ferric iron is available to complex the anion at the beginning of the experimental run (before bleeding). Fluoride concentration is high during this period because minerals, such as fluorite (present in the ore) are readily dissolved in acid media. This justifies the observance of fluoride concentrations as high as 2.4 g/L in the first 50 days of the experiment (Figure 3D). Such a situation was overcome herein by aluminum sulfate additions to the leaching solution (1 g/L Al^3+^) so that any fluoride ions quickly released from the ore would be complexed (Equations 4–7) producing species that are not capable to cross the bacterial cell membranes (Veloso et al., 2012), regardless of the kinetics of aluminum dissolution from the gangue minerals.

(4) $$\begin{array}{rcl} \text{A}{\text{l}}^{3+}+{\text{F}}^{-} & ⇆ & \text{Al}{\text{F}}^{2+}\text{ log}\beta_{1}=7.6\;\left(25^{{}^{\circ}}\text{C},\text{i}\to 0\right) \end{array}$$

(5) $${\text{Al}}^{3+}{+2\text{F}}^{\text{-}}⇆{\text{AlF}}_{\text{2}}^{+}\text{ log}\beta_{2}=12.63\;(25^{{}^{\circ}}\text{C},\text{I}\to 0)$$

(6) $$\begin{array}{rcl} \text{A}{\text{l}}^{3+}+3{\text{F}}^{-} & ⇆ & \text{Al}{\text{F}}_{3\left(\text{aq}\right)}\text{ log}\beta_{3}=16.7\;\left(25^{{}^{\circ}}\text{C},\text{i}\to 0\right) \end{array}$$

(7) $${\text{Al}}^{3+}{+3\text{F}}^{-}⇆{\text{AlF}}_{3(\text{aq})}\text{ log}\beta_{3}=16.7\;(25^{{}^{\circ}}\text{C},\text{I}\to 0)$$

**Figure 4:**
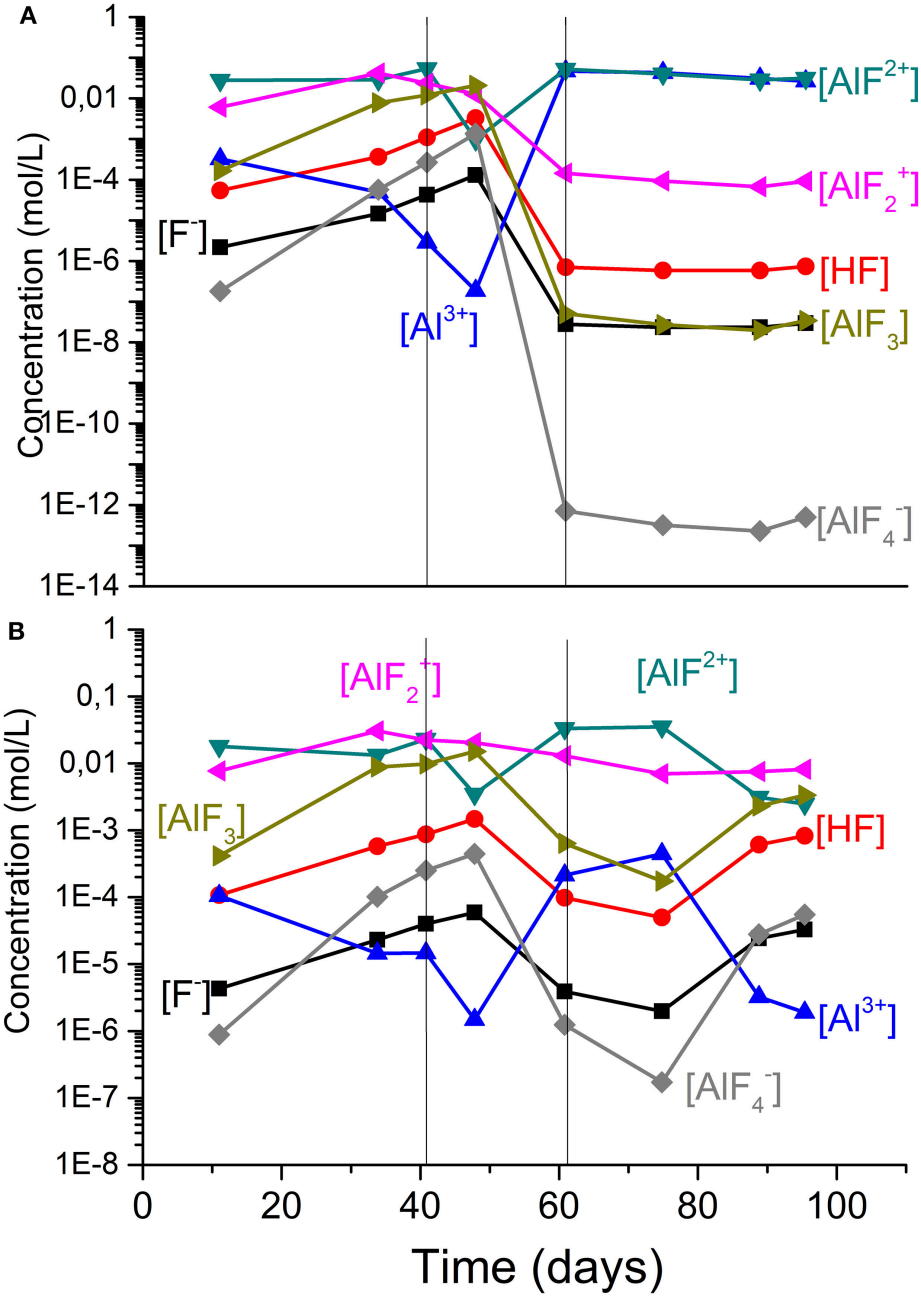
Estimated aluminium speciation in the presence of fluoride ions for high-grade ore (HG) for **(A)** the reference (C6) and **(B)** HG No-Al (C5) columns. The vertical lines indicate solution bleeding. Experiments with *S. thermosulfidooxidans* growing in Norris growth medium supplemented with yeast extract (0.1 g/L) and amended with 1.0 g/L Al^3+^; at 50 ± 1°C.

From the measured total aluminum and fluoride concentration in the PLS of each column, the distribution of aluminum-fluoride complexes was determined using equilibrium constants measured at 25°C (equilibrium constants at 50°C could not be found). Therefore, such values may be an approximation of the concentrations of such complexes at higher temperatures. Figure 4 shows the concentration of the four complexes plus HF, F^−^, and Al^3+^ for the reference column (C6) in which AlF^2+^ was the predominant aluminum fluoride complex, followed by a decrease in the HF concentration, particularly after the second bleed of the columns (60° day). These results are consistent with those previously published in a study addressing the effect of fluoride ions on ferrous iron oxidation (Veloso et al., 2012). In the column where there was no Al addition (C5), the species ${\text{AlF}}_{2}^{+}$ and AlF^2+^ were predominant, but the HF concentration was higher, highlighting the importance of aluminum supplementation in bioleaching experiments carried out in the current work.

**Figure 5:**
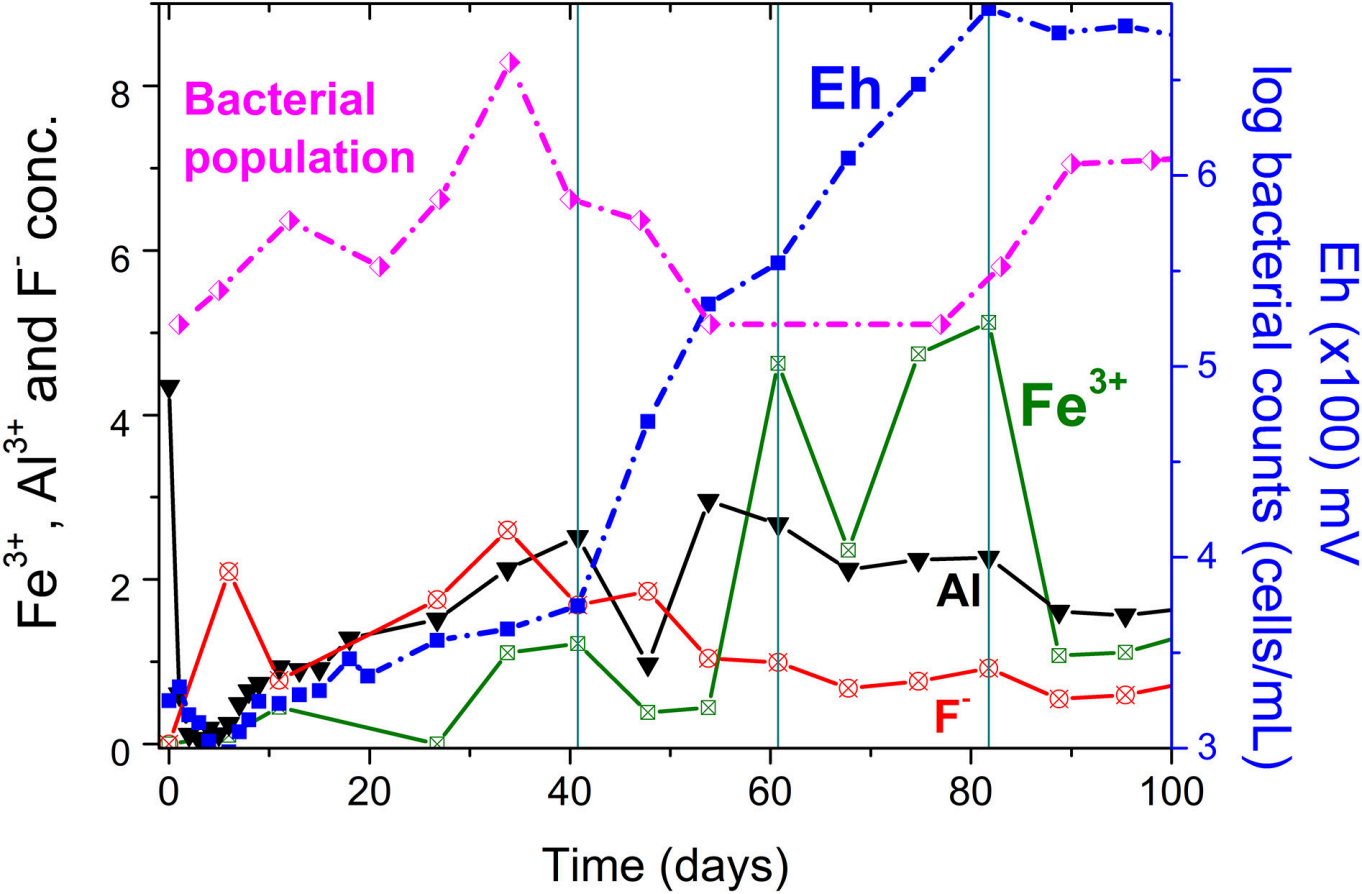
Metal and Eh profiles in the first 100 days of experiment for the reference column (high-grade ore). The vertical lines indicate solution bleeding. Experiments with *S. thermosulfidooxidans* growing in Norris growth medium supplemented with yeast extract (0.1 g/L) and amended with 1.0 g/L Al^3+^ (unless otherwise stated); at 50 ± 1°C (columns C1, C3–C6) or room temperature (column C2); crushed and agglomerated ore (¡″ top size); 8.6 L/m^2^/h solution flow-rate and 2.16 m^3^ air/m^2^/h air flow-rate.

To illustrate the combined effect of fluoride, ferric iron and aluminum on copper bioleaching, Figure 5 shows the concentration profile of these species for the reference column (C6). Before the first bleeding (41st day), a fluoride concentration (2.6 g/L) higher than that of aluminum (< 2 g/L) and a low ferric iron content (and then Eh) can be observed. Although the aluminum content appeared to provide a suitable environment for bacterial growth as the bacterial counts reached 10^6^ cells/mL before the first bleeding, the solution potential only increased after the 41st day. This lag phase was longer than that observed in the columns operating at 30°C (Rodrigues, 2013). After the bleeding, the aluminum concentration (2–3 g/L) became larger than that of fluoride (1 g/L) and with the production of ferric iron fluoride toxicity was reduced even further enabling the Eh to increase. Therefore, ferric iron reached 5 g/L in the 60° day and promoted the increase of copper extraction to 50% as compared to 20% before the first bleeding. Eventually, the solution potential attained 680 mV after 82 days and 80% copper extraction.

A parameter (η) is proposed to represent the mass ratio between total fluoride, total aluminum and total ferric iron concentrations in the system (equation 8) in order to quantify the effects of aluminum and ferric iron on fluoride toxicity. Equation (8) implies that a low η value indicates the presence of the main elements accounting for the production of fluoride complexes, thereby reduced fluoride toxicity.

(8) $$\eta =\frac{{\left[F\right]}_{\mathrm{tot}}}{{\left[\mathrm{Al}\right]}_{\mathrm{tot}}+\;{\left[Fe^{3+}\right]}_{\mathrm{tot}}}$$

**Figure 6:**
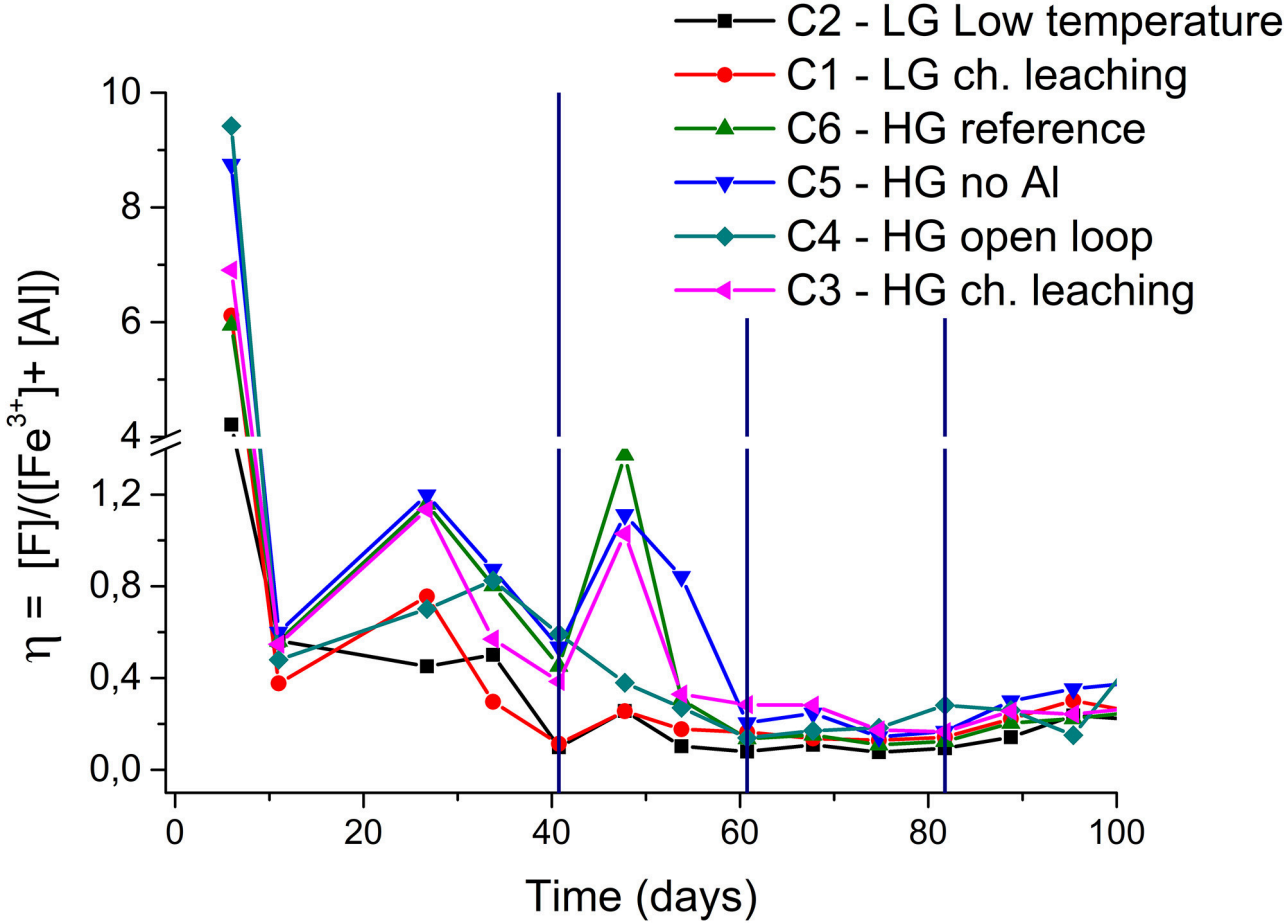
Profile of the η parameter with time during the bioleaching of inoculated columns. The vertical lines indicate solution bleeding. Experiments with *S. thermosulfidooxidans* growing in Norris growth medium supplemented with yeast extract (0.1 g/L) and amended with 1.0 g/L Al^3+^ (unless otherwise stated); at 50 ± 1°C (columns C1, C3–C6) or room temperature (column C2); crushed and agglomerated ore (¡″ top size); 8.6 L/m^2^/h solution flow-rate and 2.16 m^3^ air/m^2^/h air flow-rate.

As shown in Figure 6, the η parameter was largest during the first days of bioleaching (up to the second solution bleeding) and this was a result of a faster dissolution of fluoride-bearing minerals as compared to that of aluminum-containing compounds. That resulted in aluminum concentrations lower than that of fluoride, as already stated for the reference column (Figure 5). During this period, ferric iron concentrations were low and did not significantly contribute to reduce the HF concentration in the system. Figure 6 also indicates that even after the first bleeding, η values close to 1 were still observed in the columns C5 (*HG—no Al*) and C6 (*HG—reference)* up to the second solution bleeding (81st day). Nevertheless, although the η parameter was not affected, fluoride concentrations were reduced to values below 1.0 g/L (Figure 3D), which benefited bacterial growth. Subsequently, η was reduced to values below 0.4 (after the second bleeding) even in the column with no aluminum addition because ferric iron concentration became relevant (Eh was high) and fluoride concentration stabilized in the system (Figure 3D).

When assessing the potential for copper extraction from low-grade secondary sulfides by bioleaching, the content of both fluoride and chloride in the ore should always be determined. Once detected either element, a complete mineralogical analysis would indicate if readily soluble fluoride minerals are present so that a strategy to enable bioleaching of such ores can be defined. If fluoride is quickly dissolved and there is potential for very large fluoride concentrations as those measured by Brierley and Kuhn (2010), an acid pre-leaching step could be carried out prior to inoculating the heap as applied to control magnesium concentrations during the bioleaching of a low-grade nickel sulfide ore containing 30% MgO (Zhen et al., 2009). Nevertheless, it should be realized that fluoride is toxic at very low levels and depending on the liquor chemistry such a procedure might not be as effective. A second approach comprises adding soluble aluminum compounds, such as Al_2_(SO_4_)_3_ during column inoculation and also during the period corresponding to the fast release of fluoride from the ore as applied in the current work. For 1.0 g/L total fluoride, aluminum concentrations ~1–2 g/L would overcome fluoride toxicity without a significant impact on the solution ionic strength. As bacterial growth resulted in an increasing Eh, the produced ferric iron would also contribute to reduce the fluoride toxicity.

## Conclusions

When readily soluble fluoride-containing minerals are identified in sulfide ores, inhibitory effect in bioleaching can be forecasted. Such effects can be minimized by the addition of an external aluminum source as Al_2_(SO_4_)_3_ to the growth medium. This is because aluminum forms strong complexes with the fluoride released from the ore with positive effects on bacterial growth and producing copper extractions above 90%. Such an approach was adopted in the current work because the presence of fluorite and biotite accounted for the released of high fluoride concentrations (up to 2.5 g/L) during bioleaching of a secondary copper sulfide ore. In addition a series of solution bleedings had also positive effects on copper bioleaching throughout a decrease of both the ionic strength and fluoride concentrations.

A fluoride-toxicity parameter (η) was proposed to represent the mass ratio between total fluoride, total aluminum and total ferric iron concentrations in the system. Therefore, a low η value indicated the presence of the main elements accounting for the production of fluoride complexes and thus reduced fluoride toxicity. Values of η below 0.4 were recorded as ferric iron concentrations (which also complex fluoride, reducing the toxicity of HF) became relevant (when the Eh increased) and fluoride concentration stabilized in the system, suggesting good conditions for bacterial growth and thus bioleaching. Therefore, if the mineralogical characterization indicates the presence of fluoride minerals, a strategy should be selected to avoid any detrimental effect on bacterial growth and thus bioleaching. Solution bleeding and aluminum additions to the system can successfully accomplish such tasks.

## Author Contributions

All authors listed have made a substantial, direct and intellectual contribution to the work, and approved it for publication.

### Conflict of Interest Statement

The authors declare that the research was conducted in the absence of any commercial or financial relationships that could be construed as a potential conflict of interest.
